# D-tagatose protects against oleic acid-induced acute respiratory distress syndrome in rats by activating PTEN/PI3K/AKT pathway

**DOI:** 10.3389/fimmu.2022.928312

**Published:** 2022-09-15

**Authors:** Jian Huang, Bingjie Wang, Shaoyi Tao, Yuexia Hu, Ning Wang, Qiaoyun Zhang, Chunhui Wang, Chen Chen, Bingren Gao, Xingdong Cheng, Yongnan Li

**Affiliations:** ^1^ Department of Cardiac Surgery, Lanzhou University Second Hospital, Lanzhou University, Lanzhou, China; ^2^ Department of Anesthesiology, The Forth Affiliated Hospital of Anhui Medical University, Hefei, China; ^3^ Department of Plastic Repair Burn Surgery Dermatology, The Second People’s Hospital of Hefei, Hefei Hospital Affiliated to Anhui Medical University, Hefei, China; ^4^ Key Laboratory of Anesthesiology and Perioperative Medicine of Anhui Higher Education Institutes, Anhui Medical University, Hefei, China

**Keywords:** D-tagatose, acute respiratory distress syndrome, PTEN/PI3K/AKT pathway, oleic acid, alveolar

## Abstract

Acute respiratory distress syndrome (ARDS) is characterized by disruption of the alveolar–capillary barrier, resulting in severe alveolar edema and inflammation. D-tagatose (TAG) is a low-calorie fructose isomer with diverse biological activities whose role in ARDS has never been explored. We found that TAG protects lung tissues from injury in the oleic acid-induced rat model of ARDS. Seventeen male Sprague–Dawley rats were randomly assigned to 3 groups: Sham (n = 5), ARDS (n = 6), and TAG + ARDS (n = 6). The treatment groups were injected with oleic acid to induce ARDS, and the TAG + ARDS group was given TAG 3 days before the induction. After the treatments, the effect of TAG was evaluated by blood gas analysis and observing the gross and histological structure of the lung. The results showed that TAG significantly improved the oxygenation function, reduced the respiratory acidosis and the inflammatory response. TAG also improved the vascular permeability in ARDS rats and promoted the differentiation of alveolar type II cells, maintaining the stability of the alveolar structure. This protective effect of TAG on the lung may be achieved by activating the PTEN/PI3K/AKT pathway. Thus, TAG protects against oleic acid-induced ARDS in rats, suggesting a new clinical strategy for treating the condition.

## Introduction

ARDS is considered a significant health and economic burden. The incidence of ARDS in the intensive care units (ICUs) of 50 countries was 10.4% (1). ARDS involves a cascade of secondary inflammatory injury and secondary diffuse lung parenchymal injury mediated by various inflammatory mediators and effector cells (2–4). Despite numerous studies in recent years, the mortality of ARDS is still very high due to the complexity of etiology and pathogenesis (2). The alveolar epithelial barrier and the pulmonary microvascular endothelial barrier are the two central physiological barriers in the lung (3). During lung injury, pulmonary microvascular permeability increases from barrier damage, and protein-rich fluid exudes from the alveolar space, causing pulmonary edema and promoting the formation of hyaline membranes (2, 4). Evidence suggests that the alveolar epithelial barrier is more resistant to injury than the pulmonary microvascular endothelial (5). Therefore, promoting the repair of the alveolar epithelial barrier is crucial for improving lung injury in patients with ARDS. To seek an effective therapeutic approach to ARDS, it is important to study the pathological mechanisms of ARDS in ARDS animal model (6). Oleic acid (OA)-induced lung injury is a relevant model to study ARDS because this fatty acid acts directly on the lung cells or lung endothelium and triggers activation of different innate immune receptors (7).

The PI3K/AKT signaling pathway promotes cell growth, survival, and differentiation (8). According to previous studies, activating it protects lung epithelial cells under oxidative stress and prevents stress-induced apoptosis (9, 10). Phosphatase and tensin homolog (PTEN) is a major negative regulator of the PI3K/AKT pathway (8). It dephosphorylates PIP3 through phospholipase activity, inhibiting the pathway (11).

D-tagatose (TAG) is a rare fructose isomer and has recently been used as a new functional sweetener (12). It has a low caloric value and hypoglycemic, intestinal flora-regulating, prevent colon cancer, and lower cholesterol properties (13, 14). The heat generated is only 1/3 that of sucrose, and the energy value is 1.5 kcal/g (12). In 2001, the US Food and Drug Administration appointed TAG as a generally recognized safe food (15). It is a promising sugar substitute because it reduces the detrimental effects of fructose on the metabolic profile and the associated cardiac susceptibility to ischemia/reperfusion injury (16). In this study, the rat ARDS model induced by OA was used to evaluate the protective effect of TAG on lung tissues to provide a basis for further exploring its underlying mechanism.

## Material and methods

### Experimental animals

Seventeen male Sprague-Dawley rats (320 g ± 50 g) were randomly divided into three groups: Sham (n = 5), ARDS (n = 6), and TAG + ARDS (n = 6). They were placed in cages with a controlled temperature range (20°C–22°C) and a 12-hour light-dark cycle. The animals were given free water and food. The study protocol was approved by the Laboratory Animal Ethics Committee of Anhui Medical University (License No: LISC20180351) and performed according to the ARRIVE guidelines for animal experiments.

### Experimental protocol

**Figure 1:**
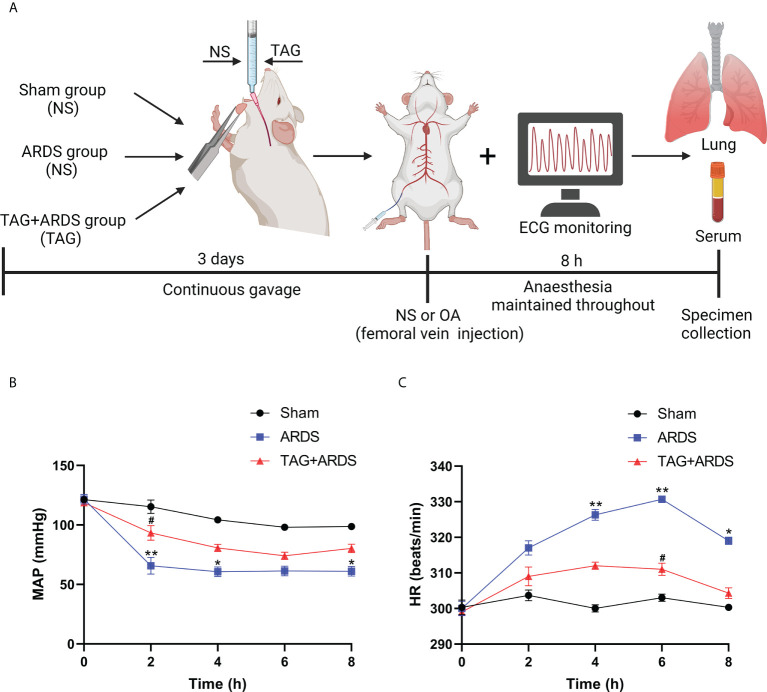
**(A)** Flow chart of the experimental procedure. **(B, C)** An ECG monitor was used to monitor MAP and HR changes throughout the experiment. Data are presented as the mean ± SD. ^*^ *p* < 0.05, ^**^ *p* < 0.01, compared with the Sham group; ^#^ *p* < 0.05, compared with the ARDS group. Illustrations were created using BioRender (biorender.com). ECG monitor, electrocardiogram monitor; TAG, D-tagatose; OA, oleic acid; NS, normal saline; ARDS, acute respiratory distress syndrome; MAP, mean arterial pressure; HR, heart rate.

TAG was purchased from Selleck (Houston, USA) and dissolved in normal saline (NS). A flow chart of the experimental procedure is shown in **Figure 1A**. The rats were anesthetized intraperitoneally with 30 mg/kg of pentobarbital, followed by separating and exposing the right femoral artery and vein. A 24-gauge catheter was catheterized into the right femoral artery to continuously monitor mean arterial pressure (MAP) and heart rate (HR) using an electrocardiogram (ECG) monitor. Those in the TAG + ARDS group were given 300 mg/kg of TAG and the 300 mg/kg of saline was given to the ARDS group by gavage for three days before OA administration. The rats in the ARDS group were treated with highly pure (99.9%) OA (Sigma, USA) by slowly injecting it into the right femoral vein with a microsyringe at a 100 mg/kg dose. The same doses of NS were provided to the Sham group. All rats were given carprofen (5 mg/kg) for postoperative analgesia. After 8 hours, the animals were euthanized with an intravenous overdose of pentobarbital. Subsequently, bronchoalveolar lavage fluid (BALF), lung tissues, and blood samples were collected for further evaluation.

### Blood gas analysis, BALF protein concentration, and Wet/Dry weight ratio

Blood samples collected from the right femoral artery were analyzed using a blood analyzer and EG7+ cartridges (Abbott, USA) to assess the oxygenation and metabolic state in each treatment group. Lungs were lavaged using 2.0 mL normal saline to collect BALF. Protein levels were quantified using a bicinchoninic acid kit (Solarbio, China). The left lung was weighed to determine the wet weight and placed in an oven at 70°C for 72 hours. When completely dehydrated, it was weighed to estimate the dry weight. Finally, the Wet/Dry weight ratio of the left lung was calculated. The oxygenation index (OI) was determined with the PaO_2_/FiO_2_ ratio.

### Enzyme-linked immunosorbent assay

The levels of TNF-α, IL-1β, IL-6, and IL-8 were quantified in the BALF and lung tissues using ELISA assay (Enzyme-linked Biotechnology, China) following the manufacturer’s instructions.

### Histopathological evaluation

The isolated lung was fixed in 4% paraformaldehyde at 4°C for 48 hours, embedded in paraffin, and sectioned at a 5 µm thickness. The sections were stained with hematoxylin-eosin, immunofluorescence, etc. For the hematoxylin–eosin staining, lung injury was determined by evaluating alveolar congestion, edema, inflammatory cell infiltration, and interstitial thickening. The lung injury score was defined as follows: none, 0; mild, 1; moderate, 2; and severe, 3. For the immunofluorescence staining, the sections were incubated with the primary antibodies: anti-surfactant protein C (anti-SPC) (1:200; Santa Cruz Biotechnology, USA), anti-aquaporin 5 (anti-AQP5) (1:4000; Abcam, USA), anti-Claudin 4 (1:200; Proteintech, China), anti-Keratin 8 (1:200; Proteintech, China) and anti-CD31 (1:200; Servicebio, China) at 4°C overnight. The images were obtained with a confocal microscope (Carl Zeiss, Germany). For the immunohistochemistry staining, the sections were incubated with an anti-myeloperoxidase (anti-MPO) primary antibody (1:2000; Abcam, USA). Histopathological evaluation was double blinded and performed by two pathologists.

### Apoptosis assay

Apoptotic cells in the lung tissue sections were quantified with TUNEL (terminal-deoxynucleotidyl transferase-mediated biotin-deoxyuridine triphosphate nick-end labeling) staining according to the manufacturer’s instructions. Five visual regions were randomly selected in each sample and recorded using a fluorescence microscope (Olympus, Japan). The TUNEL-positive rate was presented by the ratio of positively stained nuclei to total nuclei using ImageJ software (version 1.51; National Institutes of Health, USA).

### Western blot analysis

Total proteins were extracted from lung tissues using a radioimmunoprecipitation assay buffer (Beyotime, China) supplemented with protease and phosphatase inhibitors (Biosharp, China). Their levels were quantified using a bicinchoninic acid kit. Proteins were separated with sodium dodecyl sulfate–polyacrylamide gel and transferred to polyvinylidene fluoride membranes. They were blocked in 5% skim milk at 37 °C for 1 hour and incubated overnight at 4°C with primary antibodies: anti-PTEN (1:2000; Abcam, USA), anti-AKT (1:4000; Proteintech, China), anti-p-AKT (1:4000; Proteintech, China), anti-Bcl-2 (1:1000; Proteintech, China), anti-Bax (1:2000; Abcam, USA), anti-Cleaved Caspase 3 (1:2000; CST, USA), anti-SPC (1:1000; Proteintech, China), anti-AQP5 (1:1000; Proteintech, China), anti-Claudin 4 (1:4000; Proteintech, China) and anti-Keratin 8 (1:4000; Proteintech, China). β-actin (1:10000; Proteintech, China) was used as a reference protein. The membranes were incubated with a horseradish peroxidase-conjugated secondary antibody (1:10000; Proteintech, China) at 37 °C for 1 hour. Protein bands were detected with enhanced chemiluminescence detection reagents (Millipore, USA) and analyzed using ImageJ software (version 1.51; National Institutes of Health, USA).

### Reverse-transcription quantitative PCR (RT-qPCR) assay

Total RNA from lung tissues was extracted using Trizol reagent (Takara, Japan) and reversely transcribed into cDNA with a reverse transcription kit. Spectrophotometer (Thermo Fisher, USA) was used to estimate the RNA yield. The CFX96TM Real-time Detection system (Bio-RAD, USA) and TB Green qPCR Mix Plus (Takara, Japan) were used to detect mRNA levels. The PCR results were normalized to expression of β-actin. Primer sequences for PCR are shown in **Table 1**.

**Table 1:** RT-qPCR primers.

Target gene	Primer sequence
*pten*	Forward: 5′-AGGGACGAACTGGTGTAATGA-3′
	Reverse: 5′-CTGGTCCTTACTTCCCCATAGAA-3′
*pi3k*	Forward: 5′-GCCCAGGCTTACTACAGAC-3′
	Reverse: 5′-AAGTAGGGAGGCATCTCG-3′
*akt*	Forward: 5′-AGTCCCCACTCAACAACTTCT-3’
	Reverse: 5′-AAGTAGGGAGGCATCTCG-3′
*β-actin*	Forward: 5′-TGATGATATCGCCGCGCTC-3’
	Reverse: 5′-CCATCACGCCCTGGTGC-3′
*spc*	Forward: 5′-AAGAGATCCCTCTCCCAGCA-3′
	Reverse: 5′-TGGGGTTTGCCGCCATC-3′
*aqp5*	Forward: 5′-CCCTGCGGTGGTCATGAA-3′
	Reverse: 5′-CAGTCCTCCTCCGGCTCATA-3′
*claudin4*	Forward: 5′-CTCTCGCCTCCTCCACGTTACTC-3′
	Reverse: 5′-AGGGTAGGTGGGTGGGTAAG-3′
*keratin8*	Forward: 5′-CTCCGGCAGATCCATGAAGA-3′
	Reverse: 5′-GCTCGGCTGCGATTGG-3′

spc, surfactant protein C; aqp5, aquaporin 5.

### Statistical analysis

All data were presented as mean ± standard deviation (SD). To determine data normality, the Shapiro-Wilk normality test was used. The unpaired Students *t*-test was used to compare two groups with data that had a normal distribution and similar variances. For abnormal data distribution, a nonparametric test, such as the Mann-Whitney U test was used. All statistical analyses were performed using SPSS (version 22.0, California, USA) and GraphPad Prism (version 9.2.0, California, USA) software. *p* values < 0.05 were considered statistically significant.

## Results

### Perioperative hemodynamics and metabolic parameters

**Figures 1B, C** shows dynamic changes of MAP and HR throughout the experiment. MAP in the ARDS group was significantly lower than in the Sham at 2 h, 4 h and 8 h, whereas they markedly improved in the TAG + ARDS group at 2 h (TAG + ARDS *vs.* ARDS, *p* < 0.05). HR was remarkably higher in the ARDS group compared with the Sham at 4 h, 6 h and 8 h and markedly lower under the TAG pretreatment at 6 h (TAG + ARDS *vs.* ARDS, *p* < 0.05). **Table 2** summarizes the oxygenation and metabolic parameters in each group. The PaO_2_, SaO_2_, pH levels in the ARDS group were lower than in the Sham, whereas they markedly improved in the TAG + ARDS group (TAG + ARDS *vs.* ARDS, *p* < 0.001, *p* < 0.01, *p* < 0.01, respectively). The PaCO_2_ in the ARDS group was higher than in the Sham, whereas they significantly reduced in the TAG + ARDS group (TAG + ARDS *vs.* ARDS, *p* < 0.01).

**Table 2:** Metabolic changes in each group throughout the procedure.

Biochemical parameters	Sham	ARDS	TAG + ARDS
pH	7.37 ± 0.01	7.18 ± 0.02^***^	7.32 ± 0.02**^# #^**
PaCO_2_ (mmHg)	42.65 ± 2.56	64.22 ± 2.83^***^	48.09 ± 2.71**^# #^**
PaO_2_ (mmHg)	86.44 ± 1.04	29.48 ± 2.21^***^	76.40 ± 1.81**^# # #^**
SaO_2_ (%)	98.52 ± 0.91	95.49 ± 0.72^**^	97.25 ± 0.65**^# #^**
Na^+^ (mmol/l)	137.50 ± 0.88	140.40 ± 2.17	141.50 ± 2.05
K^+^ (mmol/l)	4.37 ± 0.07	4.85 ± 0.09	4.57 ± 0.21
Ca^2+^ (mmol/l)	1.39 ± 0.01	1.36 ± 0.02	1.28 ± 0.01
Hct (%)	40.18 ± 0.14	41.57 ± 0.89	40.41 ± 0.99
Hb (g/dL)	14.41 ± 0.08	14.58 ± 0.20	14.25 ± 0.09
Lac	0.56 ± 0.09	1.64 ± 0.19	1.00 ± 0.02

PaCO_2_, arterial partial pressure of CO_2_; PaO_2_, arterial partial pressure of O_2_; Hct, Hematocrit; Hb, Hemoglobin. ^**^p < 0.01, ^***^p < 0.001 versus Sham group; **^##^** p < 0.01, **^###^** p < 0.001 versus ARDS group.

### Effects of TAG on OA-induced lung injury

**Figure 2:**
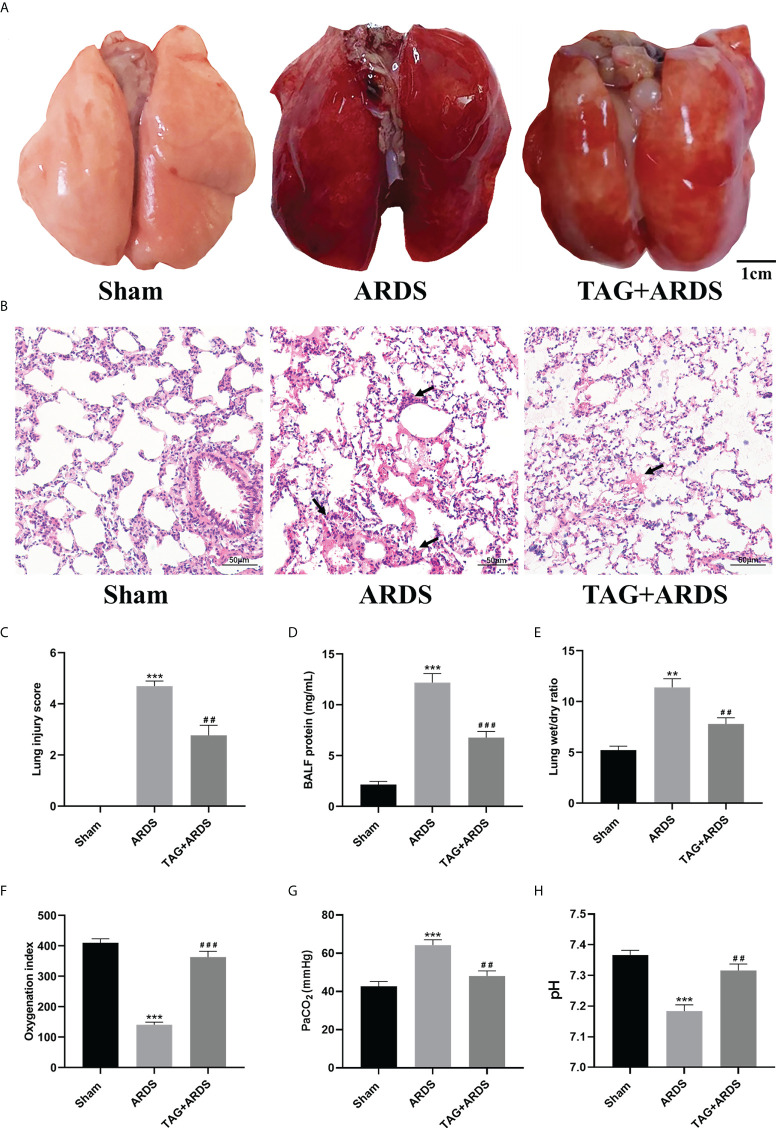
TAG pretreatment protects from OA-induced lung injury in Sprague-Dawley rats. **(A)** Gross pathological images of lung injury across experimental animal groups (scale bar = 1 cm): Sham, ARDS, and TAG+ ARDS (pretreated with TAG 3 days before ARDS induction). **(B, C)** Representative histological images of lung and lung injury score in the groups (scale bar = 50 μm). Arrows represent edema and inflammatory cell infiltration in alveolar cavity. **(D)** Protein levels in BALF in each group. **(E)** Wet/Dry weight ratio of the left lung. **(F–H)** OI, PaCO_2_ and pH determined for each group. Data are presented as the mean ± SD. ^**^ *p* < 0.01, ^***^ *p* < 0.001, compared with the Sham group; ^##^ *p* < 0.01, ^###^ *p* < 0.001, compared with the ARDS group. TAG, D-tagatose; OA, oleic acid; ARDS, acute respiratory distress syndrome; BALF, bronchoalveolar lavage fluid; OI, Oxygenation indexes.

We established a rat model of OA-induced ARDS to assess whether TAG affects lung injury. As shown in **Figure 2A**, the lung tissues in the ARDS group showed dark-red congestion, edema, and exudation compared with those in the Sham group. All three signs significantly improved under the TAG pretreatment. Moreover, histological analysis of lung tissues indicated that increased infiltration of inflammatory cells in the alveolar cavity, edema, and interstitial thickening observed in the ARDS group considerably improved in the TAG + ARDS group (**Figure 2B**). Lung injury score was also significantly lower in the TAG + ARDS group than in the ARDS (TAG + ARDS, 2.77 ± 0.40 *vs.* ARDS, 4.69 ± 0.20, *p* < 0.01; **Figure 2C**). The protein levels in BALF were higher in the ARDS group than in the Sham and were significantly reduced by the TAG pretreatment (TAG + ARDS, 6.76 ± 0.61 *vs.* ARDS, 12.18 ± 0.89, *p* < 0.001; **Figure 2D**). Similarly, the Wet/Dry ratio of lung tissues in the ARDS group was higher than that in the Sham and significantly decreased under the TAG pretreatment (TAG + ARDS, 7.80 ± 0.60 *vs.* ARDS, 11.39 ± 0.85, *p* < 0.01; **Figure 2E**). OI of all animal groups was lower than 300 after establishing the ARDS model and markedly improved in the TAG + ARDS group versus the ARDS (ARDS, 140.30 ± 8.51 *vs.* TAG + ARDS, 363.30 ± 18.56, *p* < 0.001; **Figure 2F**). The PaCO_2_ level was significantly higher in the ARDS group compared with the Sham and the TAG pretreatment decreased the level (TAG + ARDS, 48.09 ± 2.71 vs. ARDS, 64.22 ± 2.83, *p* < 0.01; **Figure 2G**). The pH level was significantly lower in the ARDS group compared with the Sham and the TAG pretreatment increased the level (ARDS, 7.18 ± 0.02 *vs.* TAG + ARDS, 7.32 ± 0.02, *p* < 0.01; **Figure 2H**).

### Effects of TAG on ARDS-induced inflammation

**Figure 3:**
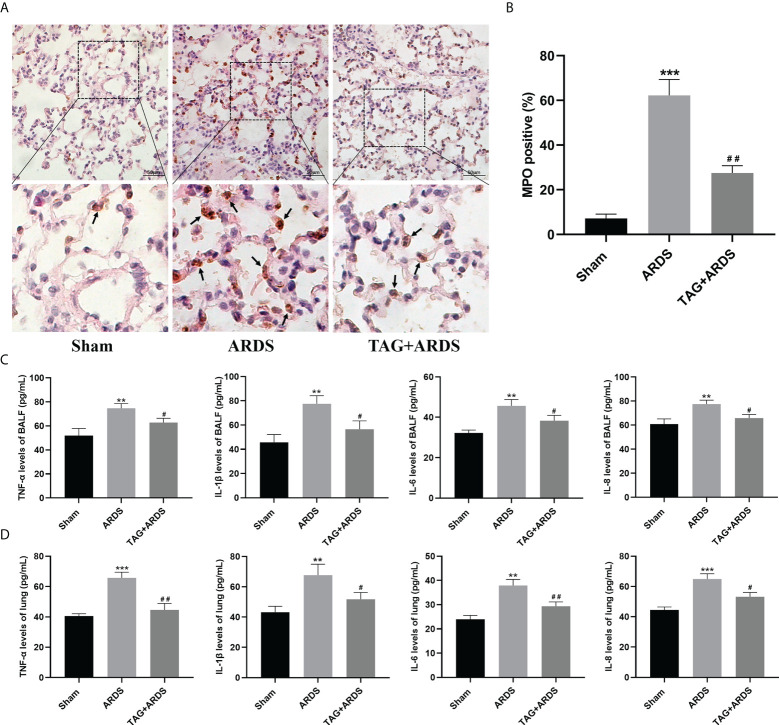
Effects of TAG on ARDS-induced inflammation. **(A, B)** Representative images of MPO-positive lung cells and their quantification in experimental animal groups: Sham, ARDS, and TAG + ARDS. Arrows represent neutrophil infiltrations (scale bar = 50 μm). **(C, D)** Levels of TNF-α, IL-1β, IL-6, and IL-8 in BALF and lung tissues assessed by ELISA. Data are presented as mean ± SD. ^**^ *p* < 0.01, ^***^ *p* < 0.001, compared with the Sham group; ^#^ *p* < 0.05, ^##^ *p* < 0.01, compared with the ARDS group. MPO, myeloperoxidase; TAG, D-tagatose; ARDS, acute respiratory distress syndrome; IHC, immunohistochemistry.

To determine whether TAG also affects ARDS-induced inflammation, we performed immunohistochemistry staining and ELISA. The results revealed that more cells were positive for the neutrophil-specific marker MPO in the ARDS group than in the Sham, and their number significantly reduced under the TAG pretreatment (TAG + ARDS, 27.49 ± 3.29 *vs*. ARDS, 62.25 ± 7.12, *p* < 0.01; **Figures 3A, B**). Furthermore, the levels of TNF-α, IL-1β, IL-6, and IL-8 in BALF and lung tissues were higher in the ARDS group than in the Sham, and the TAG pretreatment decreased their levels (TAG + ARDS *vs.* ARDS, 62.82 ± 3.53 *vs.* 74.69 ± 3.89, 56.53 ± 6.89 *vs.* 77.55 ± 6.63, 38.34 ± 2.56 *vs.* 45.68 ± 3.11, and 65.83 ± 3.05 *vs.* 77.56 ± 3.22 for the BALF, respectively, *p* < 0.05; TAG + ARDS *vs.* ARDS, 44.62 ± 4.22 *vs.* 65.76 ± 3.72, *p* < 0.01, 51.74 ± 4.52 *vs.* 67.68 ± 7.19, *p* < 0.05, 29.36 ± 1.77 *vs.* 37.94 ± 2.47, *p* < 0.01, and 53.22 ± 2.87 *vs.* 65.00 ± 3.47, *p* < 0.05 for the lung, respectively; **Figures 3C, D**).

### Effects of TAG on ARDS-induced apoptosis and PTEN/PI3K/AKT pathway

**Figure 4:**
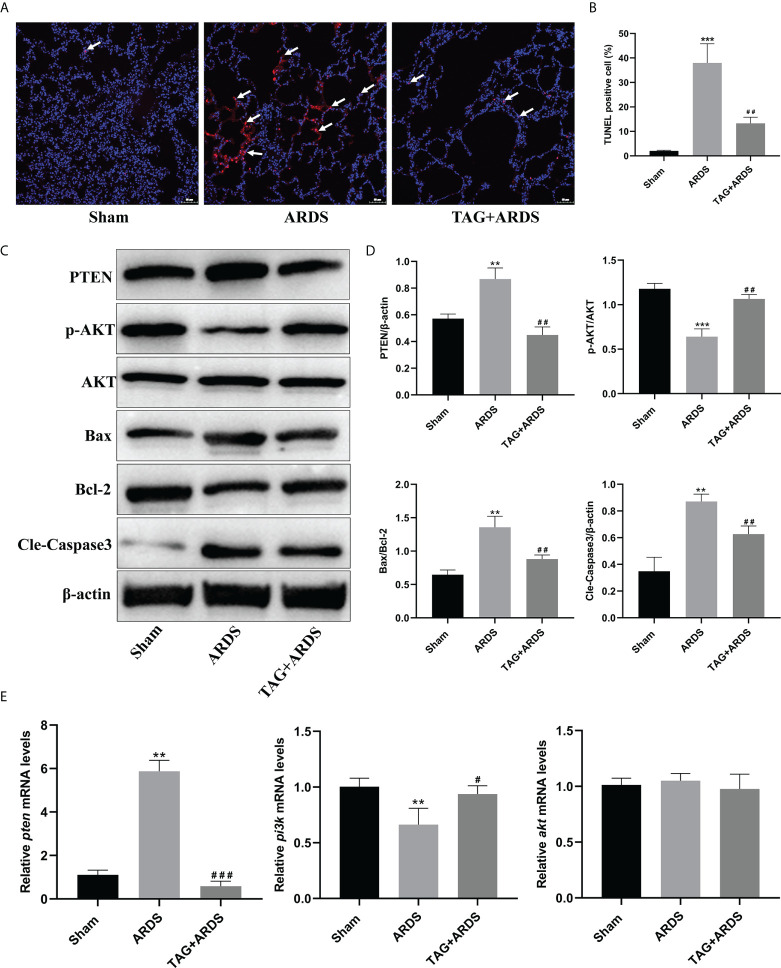
Effects of TAG on ARDS-induced apoptosis and PTEN/PI3K/AKT pathway. **(A, B)** Representative images of TUNEL-stained cells and their quantitation in lung tissue sections of 3 experimental animal groups: Sham, ARDS, and TAG + ARDS. Arrows represent apoptotic cells (scale bar = 50 μm). **(C, D)** Representative WB images and quantitation of PTEN, p-AKT/AKT, Bax/Bcl-2, and Cleaved Caspase 3 protein expression levels. **(E)** RT-qPCR results show that the transcripts levels for *pten*, *pi3k*, and *akt* mRNA. Data are presented as mean ± SD. *p* > 0.05, ^**^ *p* < 0.01, ^***^ *p* < 0.001, compared with the Sham group; *p* > 0.05, ^#^ *p* < 0.05, ^##^ *p* < 0.01, ^###^ *p* < 0.001, compared with the ARDS group. TAG, D-tagatose; ARDS, acute respiratory distress syndrome.

We also performed TUNEL staining to detect apoptotic cells in lung tissues (**Figure 4A**). We found more apoptotic cells in the ARDS group than in the Sham and observed significantly fewer apoptotic cells in the TAG + ARDS group (TAG + ARDS, 13.31 ± 2.50 *vs.* ARDS, 37.98 ± 7.85, *p* < 0.01; **Figure 4B**). In addition, we quantified the expression levels of apoptosis-related and central proteins in the PTEN/PI3K/AKT pathway with western blotting (**Figure 4C**). The expression levels of PTEN, Bax/Bcl-2 and Cleaved-Caspase3 were significantly downregulated in the TAG + ARDS group compared with the ARDS, confirming TAG relieves apoptosis (TAG + ARDS *vs.* ARDS, 0.45 ± 0.06 *vs*. 0.87 ± 0.08, 0.88 ± 0.06 *vs.* 1.36 ± 0.17, 0.63 ± 0.06 *vs.* 0.87 ± 0.05, respectively, *p* < 0.01; **Figure 4D**). Conversely, the TAG pretreatment remarkably upregulated p-AKT/AKT expression (ARDS, 0.64 ± 0.09 *vs.* TAG + ARDS, 1.06 ± 0.05, *p* < 0.01; **Figure 4D**). The RT-qPCR results show that the levels of *pten* mRNA were lower than in the TAG + ARDS group compared with the ARDS group (TAG + ARDS *vs.* ARDS, 0.59 ± 0.23 *vs.* 5.87 ± 0.51, *p* < 0.001, 0.98 ± 0.13 *vs.* 1.05 ± 0.07, *p* > 0.05, respectively; **Figure 4E**). However, TAG pretreatment markedly upregulated level of *pi3k* mRNA compared with the ARDS group (ARDS, 0.66 ± 0.15 *vs.* TAG + ARDS, 0.94 ± 0.08, *p* < 0.05; **Figure 4E**).

### Effects of TAG on ARDS-induced alveolar and endothelial damage

**Figure 5:**
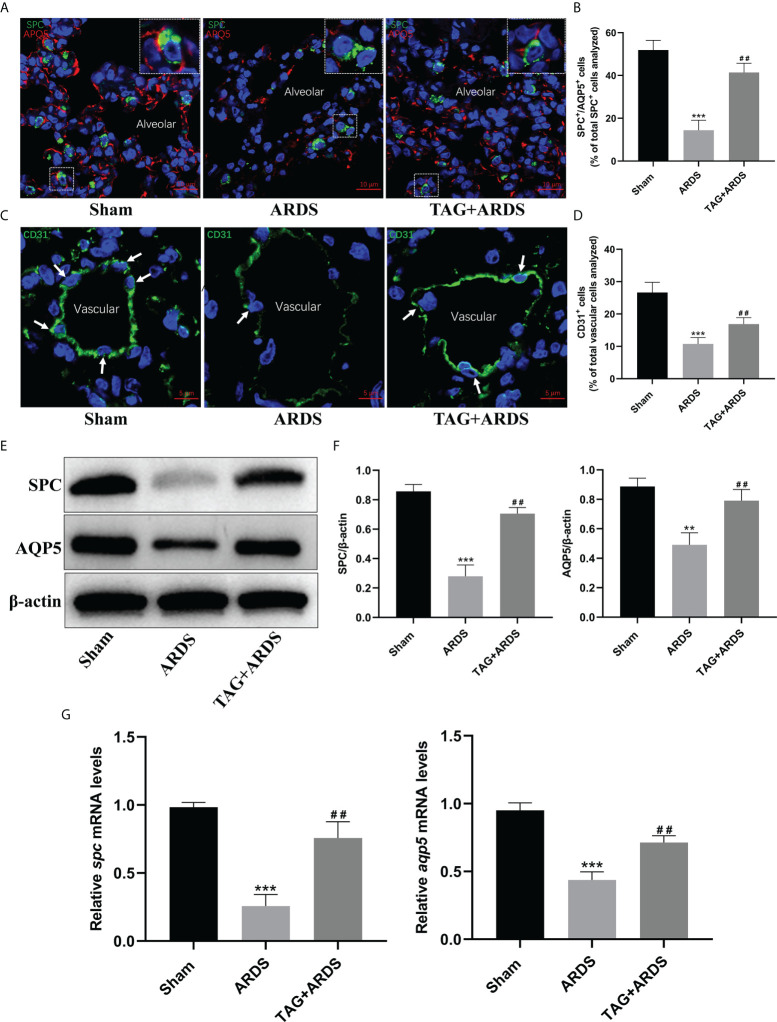
Effects of TAG on ARDS-induced alveolar and endothelial damage. **(A, B)** Representative immunofluorescence images and quantification of alveolar type I and II epithelial cells in Sham, ARDS, and TAG + ARDS groups. The lungs were co-immunostained for SPC and AQP5 alveolar epithelial-specific markers (scale bar = 10 μm). The marker-positive cells were quantified with a confocal microscope. **(C, D)** Representative immunofluorescence images and quantification of CD31-positive vascular endothelial cells in each group. Arrows represent vascular endothelial cells (scale bar = 5 μm). **(E, F)** Representative WB images and assessment of SPC and AQP5 protein expression levels in each group. **(G)** RT-qPCR results show that the transcripts levels for *spc* and *aqp5* mRNA. Data are presented as mean ± SD. ^**^ *p* < 0.01, ^***^ *p* < 0.001, compared with the Sham group; ^##^ *p* < 0.01, compared with the ARDS group. TAG, D-tagatose; ARDS, acute respiratory distress syndrome; SPC, surfactant protein C; AQP5, aquaporin 5.

**Figure 6:**
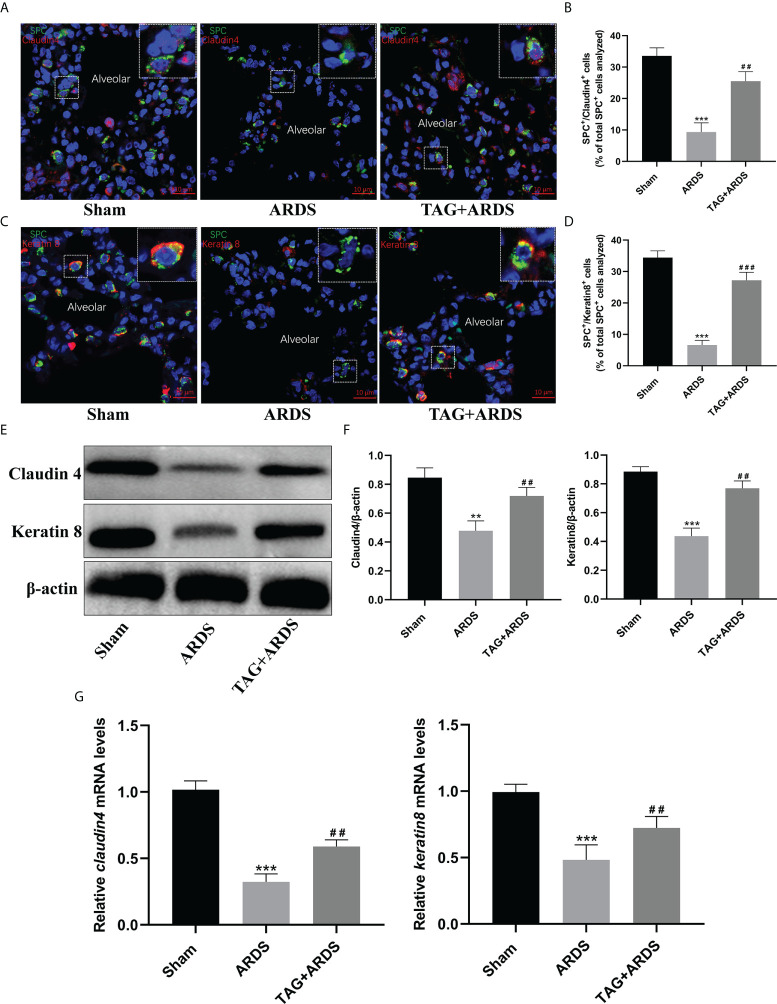
Effects of TAG on differentiation of alveolar type II cells under lung injury. **(A–D)** Representative immunofluorescence images of alveolar type II epithelial cells in the alveolar type II cells–alveolar type I cells transitional state (SPC^+^–Claudin 4^+^ and SPC^+^–Keratin 8^+^) across 3 experimental groups: Sham, ARDS, and TAG + ARDS (scale bar = 10 μm). The marker-positive cells were quantified with a confocal microscope. **(E, F)** Representative WB and quantification of Claudin 4 and Keratin 8 protein expression levels in each group. **(G)** RT-qPCR results show that the transcripts levels for *claudin 4* and *keratin 8* mRNA. Data are presented as mean ± SD. ^**^ *p* < 0.01, ^***^ *p* < 0.001, compared with the Sham group; ^##^ *p* < 0.01, ^###^ *p* < 0.001, compared with the ARDS group. TAG, D-tagatose; ARDS, acute respiratory distress syndrome; SPC, surfactant protein C.

Next, we performed immunofluorescence staining and western blotting to determine whether TAG affects the alveolar structure and the juxtaposed endothelium under inflammatory conditions. We double-stained alveolar epithelial cells for markers of alveolar type I and II cells (AQP5 and SPC) (**Figure 5A**). Results show that the expression levels of SPC and AQP5 in the ARDS group were lower than in the Sham but significantly higher on the TAG pretreatment (ARDS, 14.41 ± 4.65 *vs.* TAG + ARDS, 41.40 ± 4.33, *p* < 0.01; **Figure 5B**). We also stained vascular endothelial cells for the vascular marker CD31 and found numerous vascular endothelial cells in the TAG + ARDS group compared with the ARDS (ARDS, 10.76 ± 1.96 *vs.* TAG + ARDS, 16.88 ± 2.01, *p* < 0.01; **Figures 5C, D**). The expression levels of SPC and AQP5 proteins in the ARDS group were lower than in the Sham but increased under the TAG pretreatment (ARDS vs. TAG + ARDS, 0.28 ± 0.08 vs. 0.71 ± 0.04 and 0.49 ± 0.08 vs. 0.79 ± 0.08, respectively, p < 0.01; **Figures 5E, F**). The RT-qPCR results show that the levels of *spc*, *aqp5*, *claudin4 and keratin8* mRNA are markedly higher in the TAG + ARDS group compared with the ARDS group (ARDS *vs.* TAG + ARDS, 0.26 ± 0.09 *vs.* 0.76 ± 0.12, 0.44 ± 0.06 *vs.* 0.71 ± 0.05, 0.32 ± 0.06 *vs.* 0.59 ± 0.05, and 0.48 ± 0.11 *vs.* 0.72 ± 0.09, respectively, *p* < 0.01; **Figures 5G**, **6G**).

### Effects of TAG on differentiation of alveolar type II cells

The transitional state of alveolar type II cells was marked by Claudin 4 and Keratin 8 (**Figures 6A, C**). We identified significantly more ATII cells in the transitional state in the TAG + ARDS group than in the ARDS (ARDS *vs.* TAG + ARDS, 9.34 ± 2.94 *vs.* 25.52 ± 3.03, *p* < 0.01, 6.63 ± 1.45 *vs.* 27.23 ± 2.51, *p* < 0.001, respectively; **Figures 6B, D**). In addition, the expression levels of Claudin 4 and Keratin 8 proteins in the ARDS group were lower than in the Sham but increased under the TAG pretreatment (ARDS *vs.* TAG + ARDS, 0.48 ± 0.07 *vs.* 0.72 ± 0.06 and 0.44 ± 0.06 *vs.* 0.77 ± 0.05, respectively, *p* < 0.01; **Figures 6E, F**).

## Discussion

The specific mechanism of ARDS remains unclear, and effective drugs for treating ARDS are lacking. Thus, an OA-induced rat model of ARDS was established to explore a possible protective effect of TAG on lung injury. It is widely used in scientific research because it faithfully simulates the pathophysiological manifestations of ARDS patients and is simple, stable, and reliable (7). Subjecting the animals with OA-induced ARDS to a TAG pretreatment for 3 days significantly reduced pulmonary edema, improved gas exchange, increased the oxygenation index, reduced respiratory acidosis, and alleviated inflammation. Moreover, TAG promoted the repair of damaged alveolar epithelial and endothelial cells, inhibiting the expression of apoptosis-related proteins and activating the PTEN/PI3K/AKT pathway. These results suggest that TAG reduces OA-induced ARDS in rats, opening a new way for treating the condition.

TAG is a low-calorie sweetener and a promising novel functional food product (11). Recently, few studies explored its potential as a therapeutic drug for diseases. In one study, for example, TAG reduced the susceptibility to cardiovascular disease, but its role in respiratory diseases was unknown (15). OA induces ARDS in rats. It causes excessive protein-rich fluid exudation, extensive hyperemia, and edema, accompanied by inflammation (7). The Wet/Dry ratio of the lung is an indicator of pulmonary edema (17). We showed that TAG reduces it in OA-induced ARDS rats, which we confirmed with hematoxylin–eosin staining, among other methods. Neutrophils are crucial inflammatory cells in acute inflammation and the first-recruited cells in ARDS (18). They release proteases that cause the initial tissue damage and further migrate to the lungs for degranulation. This process releases inflammatory mediators (e.g., bactericidal proteins, cytokines, and reactive oxygen species), aggravating the inflammatory response (19, 20). Therefore, neutrophil content in alveoli reflects the degree of inflammation in the lung. We discovered that a 3-day TAG pretreatment reduces the exudation of MPO in the alveolar tissue under inflammatory conditions. This observation suggests that TAG relieves excessive ARDS-promoted secretion of inflammatory mediators in the lung. Under exacerbated pulmonary inflammation, activated alveolar macrophages release TNF-α and IL-1β, stimulating other alveolar cells (e.g., alveolar epithelial cells, macrophages, etc.) to secrete chemokines and activate the inflammatory cascade, causing continuous migration of inflammatory cells to the lungs (21). Consequently, further damage to the lung occurs. Tumor necrosis factor-alpha is a central pro-inflammatory factor in ARDS. It increases capillary permeability and initiates substantial fluid exudation, causing extensive pulmonary edema and endothelial disruption (22). Our results revealed that the expression of pro-inflammatory factors TNF-α, IL-1β, IL-6, and IL-8 in BALF and lung tissues reduces if the tissues were pretreated with TNF-α, suggesting its anti-inflammatory effect.

The PTEN/PI3K/AKT signaling pathway protects lung epithelial cells and relieves lung inflammation (23). Its activation can significantly delay the onset of acute lung injury, increasing the survival rate of animals. In humans, deleting PTEN stimulates the proliferation of malignant cells through polymorphic mutation or gene deletion, identifying the protein as a proto-oncogene (24). Although the permanent loss of PTEN function may have adverse consequences, the temporary, controllable inhibition of PTEN in lung epithelial cells is helpful for the regeneration of lung epithelium after injury (23). Moreover, the activation of PI3K/AKT signaling pathway in human lung epithelial cells occurs after PTEN is inhibited, and no agonists are later added in the process (25). We found that the PI3K/AKT signaling is inhibited in lung tissues of rats with ARDS. Pretreating the animals with TAG inhibits PTEN and activates the PI3K/AKT cascade, consistent with previous results. Thus, TAG can be used as a temporary, controllable pharmacological inhibitor of PTEN that activates the PI3K/AKT pathway and protects from lung tissue damage.

Normal alveoli are mainly composed of alveolar type I and alveolar type II cells. Alveolar type I cells are large and flat, accounting for more than 95% of the alveolar area and having an important role in gas exchange (26). Alveolar regeneration commences after acute lung injury in many mammal species. After the injury, alveolar type II cells assume stem cell properties, rapidly proliferating and differentiating into alveolar type II cells (27). Hence, they replenish the alveolar epithelium. We discovered that the number of alveolar type II cells positive increases significantly in TAG-administered rats with ARDS, suggesting enhanced proliferation of alveolar type II cells. When human and mouse lungs are injured, alveolar type II cells acquire a transient intermediate state before trans-differentiating into alveolar type I cells (28–30). Three markers for the intermediate state of alveolar type II cells are known: Claudin 4, Stratifin, and Keratin 8 (31). Interestingly, none are expressed in alveolar type II cells under physiological conditions. We found that few alveolar type II cells were in the intermediate state in lung tissues of rats with ARDS compared with control animals, indicating a compromised regenerative potential. Nonetheless, their levels increased significantly under the TAG pretreatment, suggesting that TAG promotes the repair of damaged alveoli.

Our study has several drawbacks and should be interpreted in the specific context of prophylactic administration of TAG for treating ARDS. First, it does not suggest any information on the sustained efficacy of the TAG. Second, although TAG can be administered safely over a range of doses without evident side effects, our study used only a single one (300 mg/kg) for the TAG pretreatment. Third, Oleic acid is an acid that can increase H^+^ in blood and cause acidosis and tissue damage, so it cannot completely simulate the pathophysiological process of human ARDS. Nonetheless, our data still provide valuable information for the development of drugs to treat ARDS.

## Conclusions

In conclusion, TAG pretreatment protects lung tissues from OA-induced ARDS in rats. This protective effect may be related to reducing inflammation, inhibiting apoptosis, activating the PI3K/AKT pathway, and improving alveolar and microvascular permeability. However, further experiments are necessary to identify its underlying mechanism. Our study represents the basis for developing novel therapeutic strategies for managing ARDS.

## Data availability statement

The original contributions presented in the study are included in the article/supplementary material. Further inquiries can be directed to the corresponding authors.

## Ethics statement

The animal study was reviewed and approved by the Committee of Ethics on Animal Experiments at the Anhui Medical University. Written informed consent was obtained from the owners for the participation of their animals in this study.

## Author contributions

YL, BG, and XC designed the study. JH, BW, and ST performed the experiments. YH and NW performed the statistical analysis. JH, BW and ST drafted the article. YL, BG, XC, CW and CC supervised the experimental work. All authors contributed to the article and approved the submitted version.

## Funding

This work was supported by the Basic and Clinical Cooperative Research Promotion Program of Anhui Medical University (2020xkjT046), the research fund of Anhui Medical University (2019xkj144), the Open Research fund of Key Laboratory of Anesthesiology and Perioperative Medicine of Anhui Higher Education Institutes, Anhui Medical University (MZKF202003), Lanzhou Science and Technology Bureau, science and technology planning project of Lanzhou (2020-XG-59).

## Acknowledgments

We would like to thank Home for Researchers for providing language help and writing assistance. In addition, we also thank Li Zhang and Gao Cheng for their help with the literature search and sample processing.

## Conflict of interest

The authors declare that the research was conducted in the absence of any commercial or financial relationships that could be construed as a potential conflict of interest.

## Publisher’s note

All claims expressed in this article are solely those of the authors and do not necessarily represent those of their affiliated organizations, or those of the publisher, the editors and the reviewers. Any product that may be evaluated in this article, or claim that may be made by its manufacturer, is not guaranteed or endorsed by the publisher.
